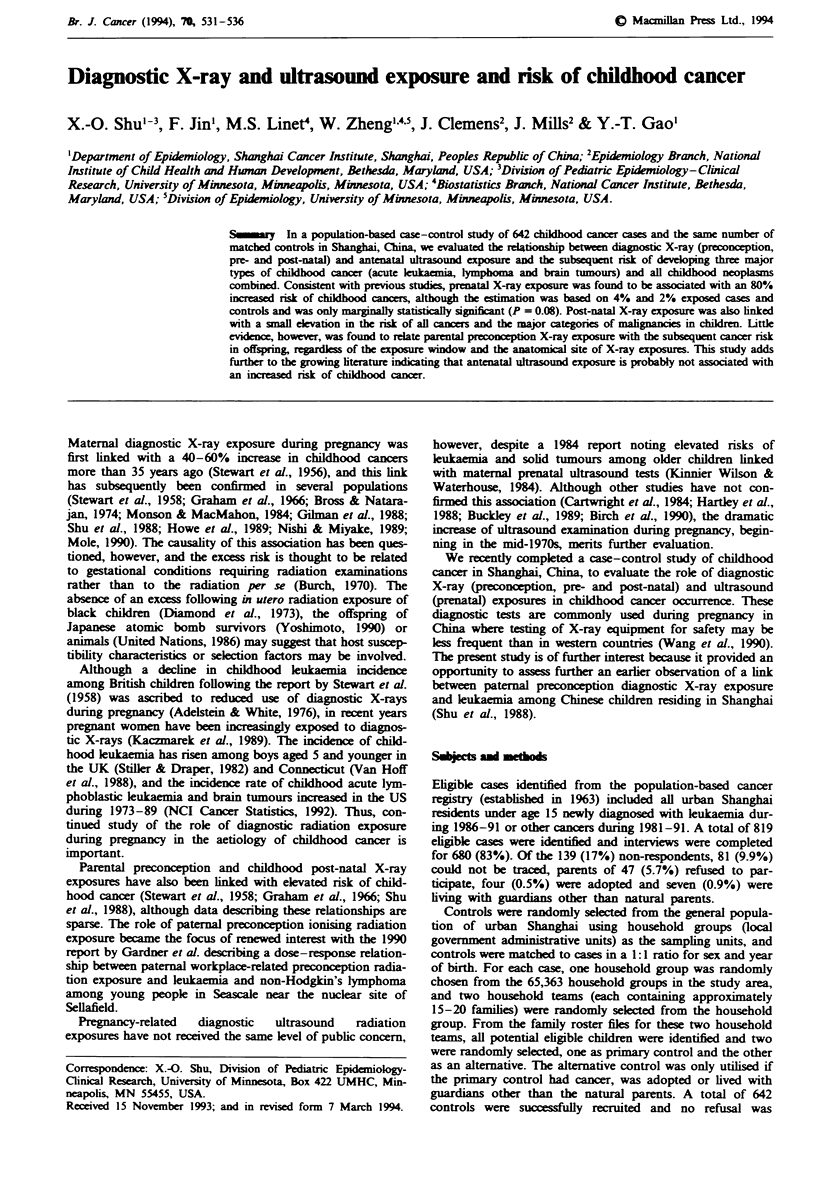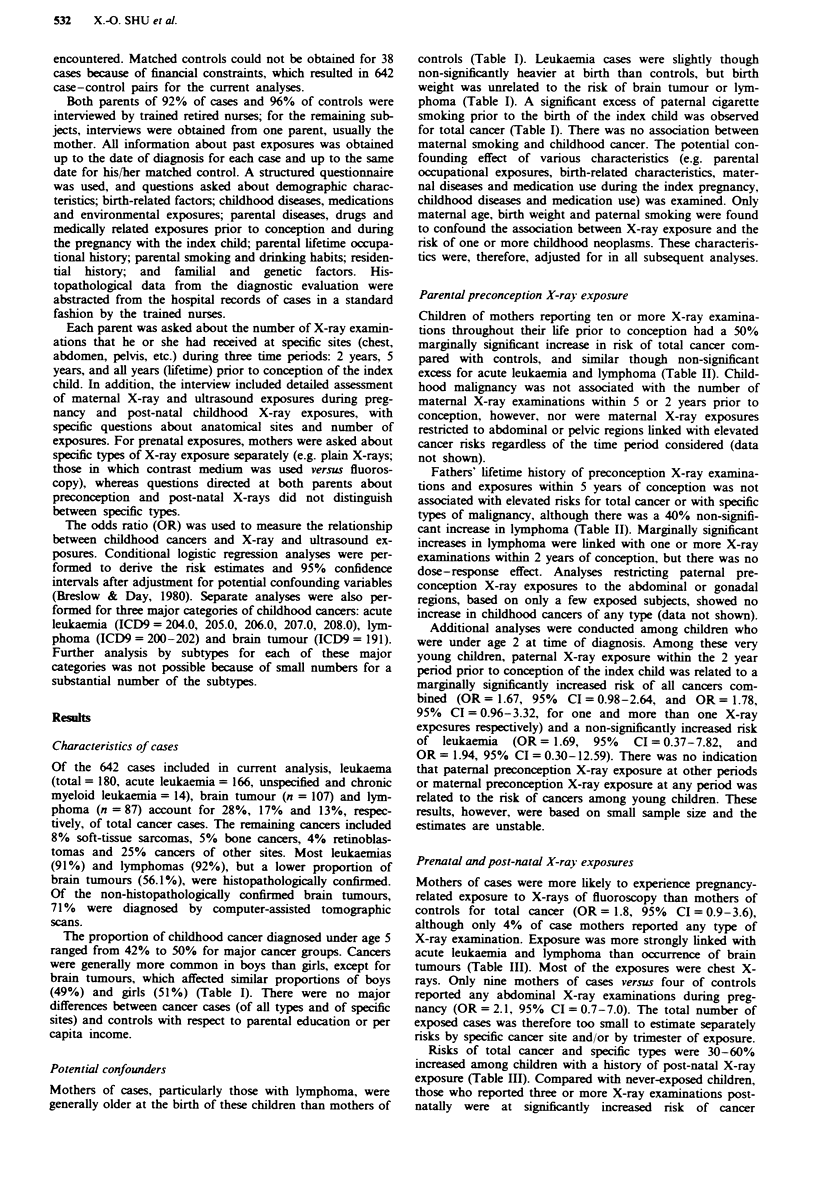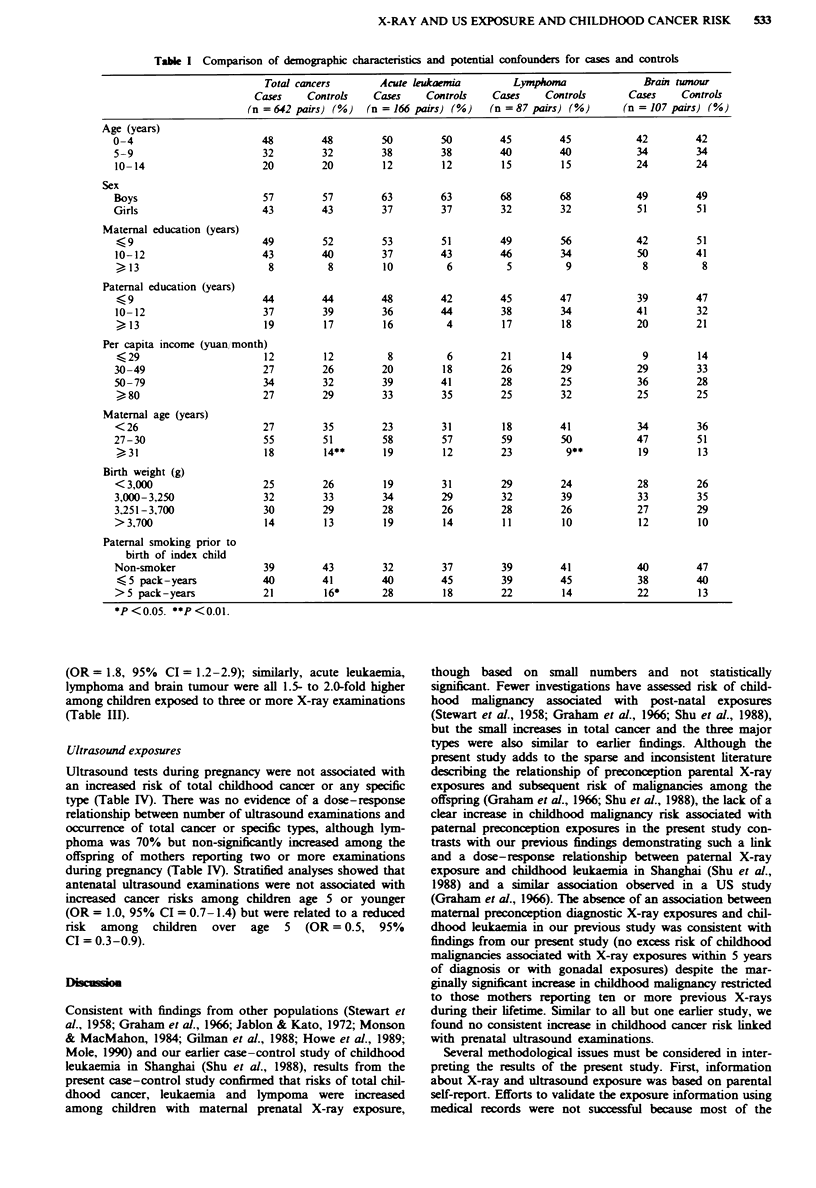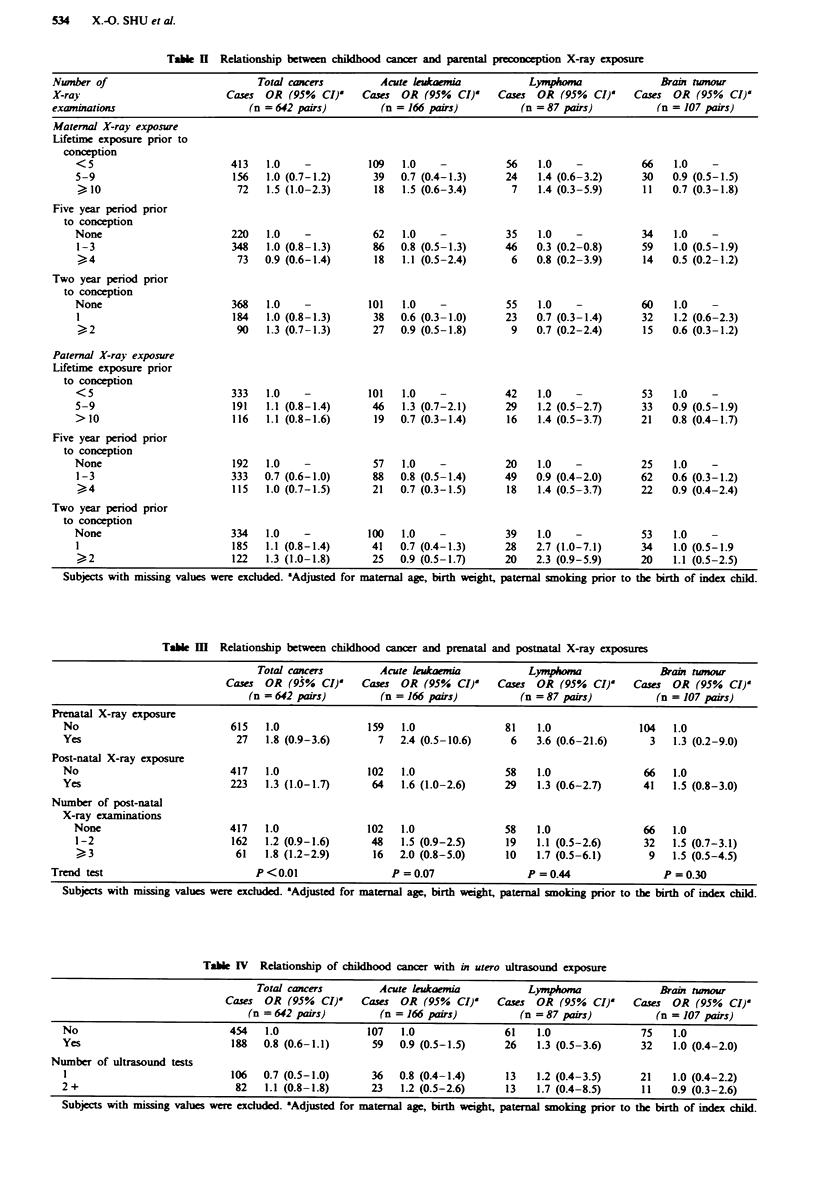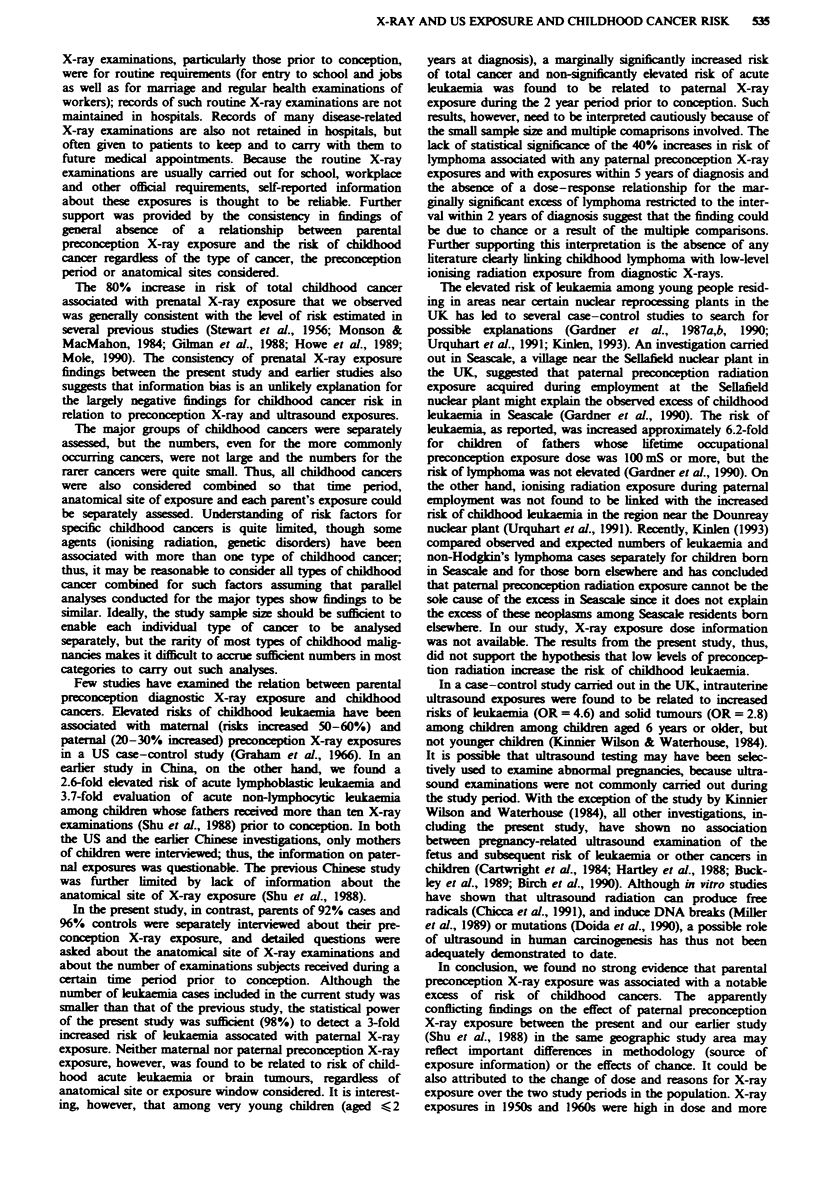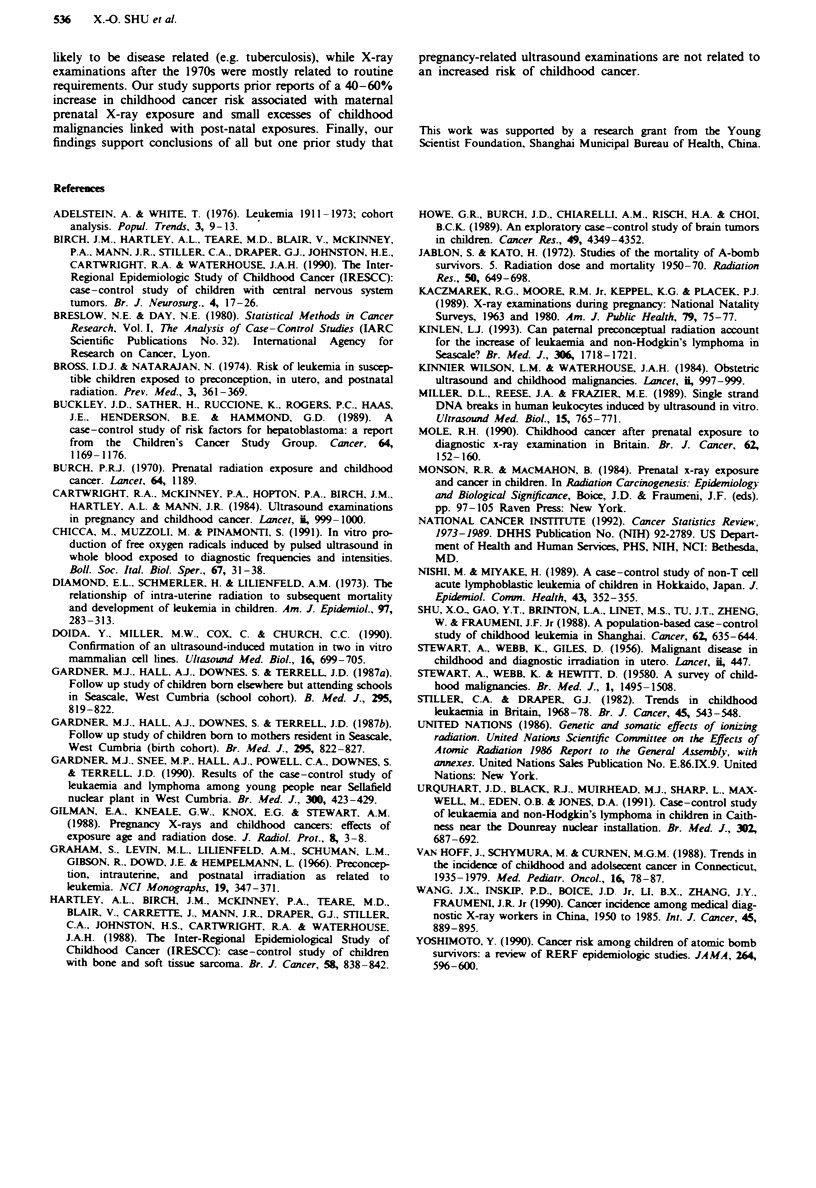# Diagnostic X-ray and ultrasound exposure and risk of childhood cancer.

**DOI:** 10.1038/bjc.1994.340

**Published:** 1994-09

**Authors:** X. O. Shu, F. Jin, M. S. Linet, W. Zheng, J. Clemens, J. Mills, Y. T. Gao

**Affiliations:** Department of Epidemiology, Shanghai Cancer Institute, Peoples Republic of China.

## Abstract

In a population-based case-control study of 642 childhood cancer cases and the same number of matched controls in Shanghai, China, we evaluated the relationship between diagnostic X-ray (preconception, pre- and post-natal) and antenatal ultrasound exposure and the subsequent risk of developing three major types of childhood cancer (acute leukaemia, lymphoma and brain tumours) and all childhood neoplasms combined. Consistent with previous studies, prenatal X-ray exposure was found to be associated with an 80% increased risk of childhood cancers, although the estimation was based on 4% and 2% exposed cases and controls and was only marginally statistically significant (P = 0.08). Post-natal X-ray exposure was also linked with a small elevation in the risk of all cancers and the major categories of malignancies in children. Little evidence, however, was found to relate parental preconception X-ray exposure with the subsequent cancer risk in offspring, regardless of the exposure window and the anatomical site of X-ray exposures. This study adds further to the growing literature indicating that antenatal ultrasound exposure is probably not associated with an increased risk of childhood cancer.


Br. J. Cancer (1994), 70, 531-536 © Macmillan Press Ltd., 1994

### Diagnostic X-ray and ultrasound exposure and risk of childhood cancer

X.-O. Shu[1-3], F. Jin[1], M.S. Linet[4], W. Zheng[1,4,5], J. Clemens[2], J. Mills[2] & Y.-T. Gao[1]

[1]*Department of Epidemiology, Shanghai Cancer Institute, Shanghai, Peoples Republic of China;* [2]*Epidemiology Branch, National Institute of Child Health and Human Development, Bethesda, Maryland, USA;* [3]*Division of Pediatric Epidemiology-Clinical Research, University of Minnesota, Minneapolis, Minnesota, USA;* [4]*Biostatistics Branch, National Cancer Institute, Bethesda, Maryland, USA;* [5]*Division of Epidemiology, University of Minnesota, Minneapolis, Minnesota, USA.*

**Summary** In a population-based case-control study of 642 childhood cancer cases and the same number of matched controls in Shanghai, China, we evaluated the relationship between diagnostic X-ray (preconception, pre- and post-natal) and antenatal ultrasound exposure and the subsequent risk of developing three major types of childhood cancer (acute leukaemia, lymphoma and brain tumours) and all childhood neoplasms combined. Consistent with previous studies, prenatal X-ray exposure was found to be associated with an 80% increased risk of childhood cancers, although the estimation was based on 4% and 2% exposed cases and controls and was only marginally statistically significant ($P = 0.08$). Post-natal X-ray exposure was also linked with a small elevation in the risk of all cancers and the major categories of malignancies in children. Little evidence, however, was found to relate parental preconception X-ray exposure with the subsequent cancer risk in offspring, regardless of the exposure window and the anatomical site of X-ray exposures. This study adds further to the growing literature indicating that antenatal ultrasound exposure is probably not associated with an increased risk of childhood cancer.

Maternal diagnostic X-ray exposure during pregnancy was first linked with a 40-60% increase in childhood cancers more than 35 years ago (Stewart *et al.*, 1956), and this link has subsequently been confirmed in several populations (Stewart *et al.*, 1958; Graham *et al.*, 1966; Bross & Natarajan, 1974; Monson & MacMahon, 1984; Gilman *et al.*, 1988; Shu *et al.*, 1988; Howe *et al.*, 1989; Nishi & Miyake, 1989; Mole, 1990). The causality of this association has been questioned, however, and the excess risk is thought to be related to gestational conditions requiring radiation examinations rather than to the radiation *per se* (Burch, 1970). The absence of an excess following *in utero* radiation exposure of black children (Diamond *et al.*, 1973), the offspring of Japanese atomic bomb survivors (Yoshimoto, 1990) or animals (United Nations, 1986) may suggest that host susceptibility characteristics or selection factors may be involved.

Although a decline in childhood leukaemia incidence among British children following the report by Stewart *et al.* (1958) was ascribed to reduced use of diagnostic X-rays during pregnancy (Adelstein & White, 1976), in recent years pregnant women have been increasingly exposed to diagnostic X-rays (Kaczmarek *et al.*, 1989). The incidence of childhood leukaemia has risen among boys aged 5 and younger in the UK (Stiller & Draper, 1982) and Connecticut (Van Hoff *et al.*, 1988), and the incidence rate of childhood acute lymphoblastic leukaemia and brain tumours increased in the US during 1973-89 (NCI Cancer Statistics, 1992). Thus, continued study of the role of diagnostic radiation exposure during pregnancy in the aetiology of childhood cancer is important.

Parental preconception and childhood post-natal X-ray exposures have also been linked with elevated risk of childhood cancer (Stewart *et al.*, 1958; Graham *et al.*, 1966; Shu *et al.*, 1988), although data describing these relationships are sparse. The role of paternal preconception ionising radiation exposure became the focus of renewed interest with the 1990 report by Gardner *et al.* describing a dose-response relationship between paternal workplace-related preconception radiation exposure and leukaemia and non-Hodgkin's lymphoma among young people in Seascale near the nuclear site of Sellafield.

Pregnancy-related diagnostic ultrasound radiation exposures have not received the same level of public concern, however, despite a 1984 report noting elevated risks of leukaemia and solid tumours among older children linked with maternal prenatal ultrasound tests (Kinnier Wilson & Waterhouse, 1984). Although other studies have not confirmed this association (Cartwright *et al.*, 1984; Hartley *et al.*, 1988; Buckley *et al.*, 1989; Birch *et al.*, 1990), the dramatic increase of ultrasound examination during pregnancy, beginning in the mid-1970s, merits further evaluation.

We recently completed a case-control study of childhood cancer in Shanghai, China, to evaluate the role of diagnostic X-ray (preconception, pre- and post-natal) and ultrasound (prenatal) exposures in childhood cancer occurrence. These diagnostic tests are commonly used during pregnancy in China where testing of X-ray equipment for safety may be less frequent than in western countries (Wang *et al.*, 1990). The present study is of further interest because it provided an opportunity to assess further an earlier observation of a link between paternal preconception diagnostic X-ray exposure and leukaemia among Chinese children residing in Shanghai (Shu *et al.*, 1988).

### Subjects and methods

Eligible cases identified from the population-based cancer registry (established in 1963) included all urban Shanghai residents under age 15 newly diagnosed with leukaemia during 1986-91 or other cancers during 1981-91. A total of 819 eligible cases were identified and interviews were completed for 680 (83%). Of the 139 (17%) non-respondents, 81 (9.9%) could not be traced, parents of 47 (5.7%) refused to participate, four (0.5%) were adopted and seven (0.9%) were living with guardians other than natural parents.

Controls were randomly selected from the general population of urban Shanghai using household groups (local government administrative units) as the sampling units, and controls were matched to cases in a 1:1 ratio for sex and year of birth. For each case, one household group was randomly chosen from the 65,363 household groups in the study area, and two household teams (each containing approximately 15-20 families) were randomly selected from the household group. From the family roster files for these two household teams, all potential eligible children were identified and two were randomly selected, one as primary control and the other as an alternative. The alternative control was only utilised if the primary control had cancer, was adopted or lived with guardians other than the natural parents. A total of 642 controls were successfully recruited and no refusal was

Correspondence: X.-O. Shu, Division of Pediatric Epidemiology-Clinical Research, University of Minnesota, Box 422 UMHC, Minneapolis, MN 55455, USA.
Received 15 November 1993; and in revised form 7 March 1994.

encountered. Matched controls could not be obtained for 38 cases because of financial constraints, which resulted in 642 case–control pairs for the current analyses.

Both parents of 92% of cases and 96% of controls were interviewed by trained retired nurses; for the remaining subjects, interviews were obtained from one parent, usually the mother. All information about past exposures was obtained up to the date of diagnosis for each case and up to the same date for his/her matched control. A structured questionnaire was used, and questions asked about demographic characteristics; birth-related factors; childhood diseases, medications and environmental exposures; parental diseases, drugs and medically related exposures prior to conception and during the pregnancy with the index child; parental lifetime occupational history; parental smoking and drinking habits; residential history; and familial and genetic factors. Histopathological data from the diagnostic evaluation were abstracted from the hospital records of cases in a standard fashion by the trained nurses.

Each parent was asked about the number of X-ray examinations that he or she had received at specific sites (chest, abdomen, pelvis, etc.) during three time periods: 2 years, 5 years, and all years (lifetime) prior to conception of the index child. In addition, the interview included detailed assessment of maternal X-ray and ultrasound exposures during pregnancy and post-natal childhood X-ray exposures, with specific questions about anatomical sites and number of exposures. For prenatal exposures, mothers were asked about specific types of X-ray exposure separately (e.g. plain X-rays; those in which contrast medium was used *versus* fluoroscopy), whereas questions directed at both parents about preconception and post-natal X-rays did not distinguish between specific types.

The odds ratio (OR) was used to measure the relationship between childhood cancers and X-ray and ultrasound exposures. Conditional logistic regression analyses were performed to derive the risk estimates and 95% confidence intervals after adjustment for potential confounding variables (Breslow & Day, 1980). Separate analyses were also performed for three major categories of childhood cancers: acute leukaemia (ICD9 = 204.0, 205.0, 206.0, 207.0, 208.0), lymphoma (ICD9 = 200–202) and brain tumour (ICD9 = 191). Further analysis by subtypes for each of these major categories was not possible because of small numbers for a substantial number of the subtypes.

### Results

### *Characteristics of cases*

Of the 642 cases included in current analysis, leukaema (total = 180, acute leukaemia = 166, unspecified and chronic myeloid leukaemia = 14), brain tumour ($n$ = 107) and lymphoma ($n$ = 87) account for 28%, 17% and 13%, respectively, of total cancer cases. The remaining cancers included 8% soft-tissue sarcomas, 5% bone cancers, 4% retinoblastomas and 25% cancers of other sites. Most leukaemias (91%) and lymphomas (92%), but a lower proportion of brain tumours (56.1%), were histopathologically confirmed. Of the non-histopathologically confirmed brain tumours, 71% were diagnosed by computer-assisted tomographic scans.

The proportion of childhood cancer diagnosed under age 5 ranged from 42% to 50% for major cancer groups. Cancers were generally more common in boys than girls, except for brain tumours, which affected similar proportions of boys (49%) and girls (51%) (Table I). There were no major differences between cancer cases (of all types and of specific sites) and controls with respect to parental education or per capita income.

### *Potential confounders*

Mothers of cases, particularly those with lymphoma, were generally older at the birth of these children than mothers of controls (Table I). Leukaemia cases were slightly though non-significantly heavier at birth than controls, but birth weight was unrelated to the risk of brain tumour or lymphoma (Table I). A significant excess of paternal cigarette smoking prior to the birth of the index child was observed for total cancer (Table I). There was no association between maternal smoking and childhood cancer. The potential confounding effect of various characteristics (e.g. parental occupational exposures, birth-related characteristics, maternal diseases and medication use during the index pregnancy, childhood diseases and medication use) was examined. Only maternal age, birth weight and paternal smoking were found to confound the association between X-ray exposure and the risk of one or more childhood neoplasms. These characteristics were, therefore, adjusted for in all subsequent analyses.

### *Parental preconception X-ray exposure*

Children of mothers reporting ten or more X-ray examinations throughout their life prior to conception had a 50% marginally significant increase in risk of total cancer compared with controls, and similar though non-significant excess for acute leukaemia and lymphoma (Table II). Childhood malignancy was not associated with the number of maternal X-ray examinations within 5 or 2 years prior to conception, however, nor were maternal X-ray exposures restricted to abdominal or pelvic regions linked with elevated cancer risks regardless of the time period considered (data not shown).

Fathers' lifetime history of preconception X-ray examinations and exposures within 5 years of conception was not associated with elevated risks for total cancer or with specific types of malignancy, although there was a 40% non-significant increase in lymphoma (Table II). Marginally significant increases in lymphoma were linked with one or more X-ray examinations within 2 years of conception, but there was no dose–response effect. Analyses restricting paternal preconception X-ray exposures to the abdominal or gonadal regions, based on only a few exposed subjects, showed no increase in childhood cancers of any type (data not shown).

Additional analyses were conducted among children who were under age 2 at time of diagnosis. Among these very young children, paternal X-ray exposure within the 2 year period prior to conception of the index child was related to a marginally significantly increased risk of all cancers combined (OR = 1.67, 95% CI = 0.98–2.64, and OR = 1.78, 95% CI = 0.96–3.32, for one and more than one X-ray exposures respectively) and a non-significantly increased risk of leukaemia (OR = 1.69, 95% CI = 0.37–7.82, and OR = 1.94, 95% CI = 0.30–12.59). There was no indication that paternal preconception X-ray exposure at other periods or maternal preconception X-ray exposure at any period was related to the risk of cancers among young children. These results, however, were based on small sample size and the estimates are unstable.

### *Prenatal and post-natal X-ray exposures*

Mothers of cases were more likely to experience pregnancy-related exposure to X-rays of fluoroscopy than mothers of controls for total cancer (OR = 1.8, 95% CI = 0.9–3.6), although only 4% of case mothers reported any type of X-ray examination. Exposure was more strongly linked with acute leukaemia and lymphoma than occurrence of brain tumours (Table III). Most of the exposures were chest X-rays. Only nine mothers of cases *versus* four of controls reported any abdominal X-ray examinations during pregnancy (OR = 2.1, 95% CI = 0.7–7.0). The total number of exposed cases was therefore too small to estimate separately risks by specific cancer site and/or by trimester of exposure.

Risks of total cancer and specific types were 30–60% increased among children with a history of post-natal X-ray exposure (Table III). Compared with never-exposed children, those who reported three or more X-ray examinations post-natally were at significantly increased risk of cancer

**Table I** Comparison of demographic characteristics and potential confounders for cases and controls

| | *Total cancers* | | *Acute leukaemia* | | *Lymphoma* | | *Brain tumour* | |
|---|---|---|---|---|---|---|---|---|
| | *Cases* | *Controls* | *Cases* | *Controls* | *Cases* | *Controls* | *Cases* | *Controls* |
| | (n = 642 pairs) (%) | | (n = 166 pairs) (%) | | (n = 87 pairs) (%) | | (n = 107 pairs) (%) | |
| Age (years) | | | | | | | | |
| 0-4 | 48 | 48 | 50 | 50 | 45 | 45 | 42 | 42 |
| 5-9 | 32 | 32 | 38 | 38 | 40 | 40 | 34 | 34 |
| 10-14 | 20 | 20 | 12 | 12 | 15 | 15 | 24 | 24 |
| Sex | | | | | | | | |
| Boys | 57 | 57 | 63 | 63 | 68 | 68 | 49 | 49 |
| Girls | 43 | 43 | 37 | 37 | 32 | 32 | 51 | 51 |
| Maternal education (years) | | | | | | | | |
| ⩽9 | 49 | 52 | 53 | 51 | 49 | 56 | 42 | 51 |
| 10-12 | 43 | 40 | 37 | 43 | 46 | 34 | 50 | 41 |
| ⩾13 | 8 | 8 | 10 | 6 | 5 | 9 | 8 | 8 |
| Paternal education (years) | | | | | | | | |
| ⩽9 | 44 | 44 | 48 | 42 | 45 | 47 | 39 | 47 |
| 10-12 | 37 | 39 | 36 | 44 | 38 | 34 | 41 | 32 |
| ⩾13 | 19 | 17 | 16 | 4 | 17 | 18 | 20 | 21 |
| Per capita income (yuan/month) | | | | | | | | |
| ⩽29 | 12 | 12 | 8 | 6 | 21 | 14 | 9 | 14 |
| 30-49 | 27 | 26 | 20 | 18 | 26 | 29 | 29 | 33 |
| 50-79 | 34 | 32 | 39 | 41 | 28 | 25 | 36 | 28 |
| ⩾80 | 27 | 29 | 33 | 35 | 25 | 32 | 25 | 25 |
| Maternal age (years) | | | | | | | | |
| <26 | 27 | 35 | 23 | 31 | 18 | 41 | 34 | 36 |
| 27-30 | 55 | 51 | 58 | 57 | 59 | 50 | 47 | 51 |
| ⩾31 | 18 | 14** | 19 | 12 | 23 | 9** | 19 | 13 |
| Birth weight (g) | | | | | | | | |
| <3,000 | 25 | 26 | 19 | 31 | 29 | 24 | 28 | 26 |
| 3,000-3,250 | 32 | 33 | 34 | 29 | 32 | 39 | 33 | 35 |
| 3,251-3,700 | 30 | 29 | 28 | 26 | 28 | 26 | 27 | 29 |
| >3,700 | 14 | 13 | 19 | 14 | 11 | 10 | 12 | 10 |
| Paternal smoking prior to birth of index child | | | | | | | | |
| Non-smoker | 39 | 43 | 32 | 37 | 39 | 41 | 40 | 47 |
| ⩽5 pack-years | 40 | 41 | 40 | 45 | 39 | 45 | 38 | 40 |
| >5 pack-years | 21 | 16* | 28 | 18 | 22 | 14 | 22 | 13 |

*$P < 0.05$. **$P < 0.01$.

(OR = 1.8, 95% CI = 1.2–2.9); similarly, acute leukaemia, lymphoma and brain tumour were all 1.5- to 2.0-fold higher among children exposed to three or more X-ray examinations (Table III).

*Ultrasound exposures*

Ultrasound tests during pregnancy were not associated with an increased risk of total childhood cancer or any specific type (Table IV). There was no evidence of a dose–response relationship between number of ultrasound examinations and occurrence of total cancer or specific types, although lymphoma was 70% but non-significantly increased among the offspring of mothers reporting two or more examinations during pregnancy (Table IV). Stratified analyses showed that antenatal ultrasound examinations were not associated with increased cancer risks among children age 5 or younger (OR = 1.0, 95% CI = 0.7–1.4) but were related to a reduced risk among children over age 5 (OR = 0.5, 95% CI = 0.3–0.9).

### Discussion

Consistent with findings from other populations (Stewart *et al.*, 1958; Graham *et al.*, 1966; Jablon & Kato, 1972; Monson & MacMahon, 1984; Gilman *et al.*, 1988; Howe *et al.*, 1989; Mole, 1990) and our earlier case–control study of childhood leukaemia in Shanghai (Shu *et al.*, 1988), results from the present case–control study confirmed that risks of total childhood cancer, leukaemia and lympoma were increased among children with maternal prenatal X-ray exposure, though based on small numbers and not statistically significant. Fewer investigations have assessed risk of childhood malignancy associated with post-natal exposures (Stewart *et al.*, 1958; Graham *et al.*, 1966; Shu *et al.*, 1988), but the small increases in total cancer and the three major types were also similar to earlier findings. Although the present study adds to the sparse and inconsistent literature describing the relationship of preconception parental X-ray exposures and subsequent risk of malignancies among the offspring (Graham *et al.*, 1966; Shu *et al.*, 1988), the lack of a clear increase in childhood malignancy risk associated with paternal preconception exposures in the present study contrasts with our previous findings demonstrating such a link and a dose–response relationship between paternal X-ray exposure and childhood leukaemia in Shanghai (Shu *et al.*, 1988) and a similar association observed in a US study (Graham *et al.*, 1966). The absence of an association between maternal preconception diagnostic X-ray exposures and childhood leukaemia in our previous study was consistent with findings from our present study (no excess risk of childhood malignancies associated with X-ray exposures within 5 years of diagnosis or with gonadal exposures) despite the marginally significant increase in childhood malignancy restricted to those mothers reporting ten or more previous X-rays during their lifetime. Similar to all but one earlier study, we found no consistent increase in childhood cancer risk linked with prenatal ultrasound examinations.

Several methodological issues must be considered in interpreting the results of the present study. First, information about X-ray and ultrasound exposure was based on parental self-report. Efforts to validate the exposure information using medical records were not successful because most of the

**Table II** Relationship between childhood cancer and parental preconception X-ray exposure

| *Number of X-ray examinations* | *Total cancers* Cases | OR (95% CI)[a] (n = 642 pairs) | *Acute leukaemia* Cases | OR (95% CI)[a] (n = 166 pairs) | *Lymphoma* Cases | OR (95% CI)[a] (n = 87 pairs) | *Brain tumour* Cases | OR (95% CI)[a] (n = 107 pairs) |
|---|---|---|---|---|---|---|---|---|
| *Maternal X-ray exposure* | | | | | | | | |
| Lifetime exposure prior to conception | | | | | | | | |
| <5 | 413 | 1.0 – | 109 | 1.0 – | 56 | 1.0 – | 66 | 1.0 – |
| 5–9 | 156 | 1.0 (0.7–1.2) | 39 | 0.7 (0.4–1.3) | 24 | 1.4 (0.6–3.2) | 30 | 0.9 (0.5–1.5) |
| ⩾10 | 72 | 1.5 (1.0–2.3) | 18 | 1.5 (0.6–3.4) | 7 | 1.4 (0.3–5.9) | 11 | 0.7 (0.3–1.8) |
| Five year period prior to conception | | | | | | | | |
| None | 220 | 1.0 – | 62 | 1.0 – | 35 | 1.0 – | 34 | 1.0 – |
| 1–3 | 348 | 1.0 (0.8–1.3) | 86 | 0.8 (0.5–1.3) | 46 | 0.3 (0.2–0.8) | 59 | 1.0 (0.5–1.9) |
| ⩾4 | 73 | 0.9 (0.6–1.4) | 18 | 1.1 (0.5–2.4) | 6 | 0.8 (0.2–3.9) | 14 | 0.5 (0.2–1.2) |
| Two year period prior to conception | | | | | | | | |
| None | 368 | 1.0 – | 101 | 1.0 – | 55 | 1.0 – | 60 | 1.0 – |
| 1 | 184 | 1.0 (0.8–1.3) | 38 | 0.6 (0.3–1.0) | 23 | 0.7 (0.3–1.4) | 32 | 1.2 (0.6–2.3) |
| ⩾2 | 90 | 1.3 (0.7–1.3) | 27 | 0.9 (0.5–1.8) | 9 | 0.7 (0.2–2.4) | 15 | 0.6 (0.3–1.2) |
| *Paternal X-ray exposure* | | | | | | | | |
| Lifetime exposure prior to conception | | | | | | | | |
| <5 | 333 | 1.0 – | 101 | 1.0 – | 42 | 1.0 – | 53 | 1.0 – |
| 5–9 | 191 | 1.1 (0.8–1.4) | 46 | 1.3 (0.7–2.1) | 29 | 1.2 (0.5–2.7) | 33 | 0.9 (0.5–1.9) |
| >10 | 116 | 1.1 (0.8–1.6) | 19 | 0.7 (0.3–1.4) | 16 | 1.4 (0.5–3.7) | 21 | 0.8 (0.4–1.7) |
| Five year period prior to conception | | | | | | | | |
| None | 192 | 1.0 – | 57 | 1.0 – | 20 | 1.0 – | 25 | 1.0 – |
| 1–3 | 333 | 0.7 (0.6–1.0) | 88 | 0.8 (0.5–1.4) | 49 | 0.9 (0.4–2.0) | 62 | 0.6 (0.3–1.2) |
| ⩾4 | 115 | 1.0 (0.7–1.5) | 21 | 0.7 (0.3–1.5) | 18 | 1.4 (0.5–3.7) | 22 | 0.9 (0.4–2.4) |
| Two year period prior to conception | | | | | | | | |
| None | 334 | 1.0 – | 100 | 1.0 – | 39 | 1.0 – | 53 | 1.0 – |
| 1 | 185 | 1.1 (0.8–1.4) | 41 | 0.7 (0.4–1.3) | 28 | 2.7 (1.0–7.1) | 34 | 1.0 (0.5–1.9 |
| ⩾2 | 122 | 1.3 (1.0–1.8) | 25 | 0.9 (0.5–1.7) | 20 | 2.3 (0.9–5.9) | 20 | 1.1 (0.5–2.5) |

Subjects with missing values were excluded. [a]Adjusted for maternal age, birth weight, paternal smoking prior to the birth of index child.

**Table III** Relationship between childhood cancer and prenatal and postnatal X-ray exposures

| | *Total cancers* Cases | OR (95% CI)[a] (n = 642 pairs) | *Acute leukaemia* Cases | OR (95% CI)[a] (n = 166 pairs) | *Lymphoma* Cases | OR (95% CI)[a] (n = 87 pairs) | *Brain tumour* Cases | OR (95% CI)[a] (n = 107 pairs) |
|---|---|---|---|---|---|---|---|---|
| Prenatal X-ray exposure | | | | | | | | |
| No | 615 | 1.0 | 159 | 1.0 | 81 | 1.0 | 104 | 1.0 |
| Yes | 27 | 1.8 (0.9–3.6) | 7 | 2.4 (0.5–10.6) | 6 | 3.6 (0.6–21.6) | 3 | 1.3 (0.2–9.0) |
| Post-natal X-ray exposure | | | | | | | | |
| No | 417 | 1.0 | 102 | 1.0 | 58 | 1.0 | 66 | 1.0 |
| Yes | 223 | 1.3 (1.0–1.7) | 64 | 1.6 (1.0–2.6) | 29 | 1.3 (0.6–2.7) | 41 | 1.5 (0.8–3.0) |
| Number of post-natal X-ray examinations | | | | | | | | |
| None | 417 | 1.0 | 102 | 1.0 | 58 | 1.0 | 66 | 1.0 |
| 1–2 | 162 | 1.2 (0.9–1.6) | 48 | 1.5 (0.9–2.5) | 19 | 1.1 (0.5–2.6) | 32 | 1.5 (0.7–3.1) |
| ⩾3 | 61 | 1.8 (1.2–2.9) | 16 | 2.0 (0.8–5.0) | 10 | 1.7 (0.5–6.1) | 9 | 1.5 (0.5–4.5) |
| Trend test | | $P<0.01$ | | $P=0.07$ | | $P=0.44$ | | $P=0.30$ |

Subjects with missing values were excluded. [a]Adjusted for maternal age, birth weight, paternal smoking prior to the birth of index child.

**Table IV** Relationship of childhood cancer with *in utero* ultrasound exposure

| | *Total cancers* Cases | OR (95% CI)[a] (n = 642 pairs) | *Acute leukaemia* Cases | OR (95% CI)[a] (n = 166 pairs) | *Lymphoma* Cases | OR (95% CI)[a] (n = 87 pairs) | *Brain tumour* Cases | OR (95% CI)[a] (n = 107 pairs) |
|---|---|---|---|---|---|---|---|---|
| No | 454 | 1.0 | 107 | 1.0 | 61 | 1.0 | 75 | 1.0 |
| Yes | 188 | 0.8 (0.6–1.1) | 59 | 0.9 (0.5–1.5) | 26 | 1.3 (0.5–3.6) | 32 | 1.0 (0.4–2.0) |
| Number of ultrasound tests | | | | | | | | |
| 1 | 106 | 0.7 (0.5–1.0) | 36 | 0.8 (0.4–1.4) | 13 | 1.2 (0.4–3.5) | 21 | 1.0 (0.4–2.2) |
| 2+ | 82 | 1.1 (0.8–1.8) | 23 | 1.2 (0.5–2.6) | 13 | 1.7 (0.4–8.5) | 11 | 0.9 (0.3–2.6) |

Subjects with missing values were excluded. [a]Adjusted for maternal age, birth weight, paternal smoking prior to the birth of index child.

X-ray examinations, particularly those prior to conception, were for routine requirements (for entry to school and jobs as well as for marriage and regular health examinations of workers); records of such routine X-ray examinations are not maintained in hospitals. Records of many disease-related X-ray examinations are also not retained in hospitals, but often given to patients to keep and to carry with them to future medical appointments. Because the routine X-ray examinations are usually carried out for school, workplace and other official requirements, self-reported information about these exposures is thought to be reliable. Further support was provided by the consistency in findings of general absence of a relationship between parental preconception X-ray exposure and the risk of childhood cancer regardless of the type of cancer, the preconception period or anatomical sites considered.

The 80% increase in risk of total childhood cancer associated with prenatal X-ray exposure that we observed was generally consistent with the level of risk estimated in several previous studies (Stewart *et al.*, 1956; Monson & MacMahon, 1984; Gilman *et al.*, 1988; Howe *et al.*, 1989; Mole, 1990). The consistency of prenatal X-ray exposure findings between the present study and earlier studies also suggests that information bias is an unlikely explanation for the largely negative findings for childhood cancer risk in relation to preconception X-ray and ultrasound exposures.

The major groups of childhood cancers were separately assessed, but the numbers, even for the more commonly occurring cancers, were not large and the numbers for the rarer cancers were quite small. Thus, all childhood cancers were also considered combined so that time period, anatomical site of exposure and each parent's exposure could be separately assessed. Understanding of risk factors for specific childhood cancers is quite limited, though some agents (ionising radiation, genetic disorders) have been associated with more than one type of childhood cancer; thus, it may be reasonable to consider all types of childhood cancer combined for such factors assuming that parallel analyses conducted for the major types show findings to be similar. Ideally, the study sample size should be sufficient to enable each individual type of cancer to be analysed separately, but the rarity of most types of childhood malignancies makes it difficult to accrue sufficient numbers in most categories to carry out such analyses.

Few studies have examined the relation between parental preconception diagnostic X-ray exposure and childhood cancers. Elevated risks of childhood leukaemia have been associated with maternal (risks increased 50–60%) and paternal (20–30% increased) preconception X-ray exposures in a US case–control study (Graham *et al.*, 1966). In an earlier study in China, on the other hand, we found a 2.6-fold elevated risk of acute lymphoblastic leukaemia and 3.7-fold evaluation of acute non-lymphocytic leukaemia among children whose fathers received more than ten X-ray examinations (Shu *et al.*, 1988) prior to conception. In both the US and the earlier Chinese investigations, only mothers of children were interviewed; thus, the information on paternal exposures was questionable. The previous Chinese study was further limited by lack of information about the anatomical site of X-ray exposure (Shu *et al.*, 1988).

In the present study, in contrast, parents of 92% cases and 96% controls were separately interviewed about their preconception X-ray exposure, and detailed questions were asked about the anatomical site of X-ray examinations and about the number of examinations subjects received during a certain time period prior to conception. Although the number of leukaemia cases included in the current study was smaller than that of the previous study, the statistical power of the present study was sufficient (98%) to detect a 3-fold increased risk of leukaemia assocated with paternal X-ray exposure. Neither maternal nor paternal preconception X-ray exposure, however, was found to be related to risk of childhood acute leukaemia or brain tumours, regardless of anatomical site or exposure window considered. It is interesting, however, that among very young children (aged $\leqslant 2$ years at diagnosis), a marginally significantly increased risk of total cancer and non-significantly elevated risk of acute leukaemia was found to be related to paternal X-ray exposure during the 2 year period prior to conception. Such results, however, need to be interpreted cautiously because of the small sample size and multiple comaprisons involved. The lack of statistical significance of the 40% increases in risk of lymphoma associated with any paternal preconception X-ray exposures and with exposures within 5 years of diagnosis and the absence of a dose–response relationship for the marginally significant excess of lymphoma restricted to the interval within 2 years of diagnosis suggest that the finding could be due to chance or a result of the multiple comparisons. Further supporting this interpretation is the absence of any literature clearly linking childhood lymphoma with low-level ionising radiation exposure from diagnostic X-rays.

The elevated risk of leukaemia among young people residing in areas near certain nuclear reprocessing plants in the UK has led to several case–control studies to search for possible explanations (Gardner *et al.*, 1987*a*,*b*, 1990; Urquhart *et al.*, 1991; Kinlen, 1993). An investigation carried out in Seascale, a village near the Sellafield nuclear plant in the UK, suggested that paternal preconception radiation exposure acquired during employment at the Sellafield nuclear plant might explain the observed excess of childhood leukaemia in Seascale (Gardner *et al.*, 1990). The risk of leukaemia, as reported, was increased approximately 6.2-fold for children of fathers whose lifetime occupational preconception exposure dose was 100 mS or more, but the risk of lymphoma was not elevated (Gardner *et al.*, 1990). On the other hand, ionising radiation exposure during paternal employment was not found to be linked with the increased risk of childhood leukaemia in the region near the Dounreay nuclear plant (Urquhart *et al.*, 1991). Recently, Kinlen (1993) compared observed and expected numbers of leukaemia and non-Hodgkin's lymphoma cases separately for children born in Seascale and for those born elsewhere and has concluded that paternal preconception radiation exposure cannot be the sole cause of the excess in Seascale since it does not explain the excess of these neoplasms among Seascale residents born elsewhere. In our study, X-ray exposure dose information was not available. The results from the present study, thus, did not support the hypothesis that low levels of preconception radiation increase the risk of childhood leukaemia.

In a case–control study carried out in the UK, intrauterine ultrasound exposures were found to be related to increased risks of leukaemia (OR = 4.6) and solid tumours (OR = 2.8) among children among children aged 6 years or older, but not younger children (Kinnier Wilson & Waterhouse, 1984). It is possible that ultrasound testing may have been selectively used to examine abnormal pregnancies, because ultrasound examinations were not commonly carried out during the study period. With the exception of the study by Kinnier Wilson and Waterhouse (1984), all other investigations, including the present study, have shown no association between pregnancy-related ultrasound examination of the fetus and subsequent risk of leukaemia or other cancers in children (Cartwright *et al.*, 1984; Hartley *et al.*, 1988; Buckley *et al.*, 1989; Birch *et al.*, 1990). Although *in vitro* studies have shown that ultrasound radiation can produce free radicals (Chicca *et al.*, 1991), and induce DNA breaks (Miller *et al.*, 1989) or mutations (Doida *et al.*, 1990), a possible role of ultrasound in human carcinogenesis has thus not been adequately demonstrated to date.

In conclusion, we found no strong evidence that parental preconception X-ray exposure was associated with a notable excess of risk of childhood cancers. The apparently conflicting findings on the effect of paternal preconception X-ray exposure between the present and our earlier study (Shu *et al.*, 1988) in the same geographic study area may reflect important differences in methodology (source of exposure information) or the effects of chance. It could be also attributed to the change of dose and reasons for X-ray exposure over the two study periods in the population. X-ray exposures in 1950s and 1960s were high in dose and more likely to be disease related (e.g. tuberculosis), while X-ray examinations after the 1970s were mostly related to routine requirements. Our study supports prior reports of a 40-60% increase in childhood cancer risk associated with maternal prenatal X-ray exposure and small excesses of childhood malignancies linked with post-natal exposures. Finally, our findings support conclusions of all but one prior study that pregnancy-related ultrasound examinations are not related to an increased risk of childhood cancer.

This work was supported by a research grant from the Young Scientist Foundation, Shanghai Municipal Bureau of Health, China.

**References**

ADELSTEIN, A. & WHITE, T. (1976). Leukemia 1911-1973; cohort analysis. *Popul. Trends*, **3**, 9-13.

BIRCH, J.M., HARTLEY, A.L., TEARE, M.D., BLAIR, V., MCKINNEY, P.A., MANN, J.R., STILLER, C.A., DRAPER, G.J., JOHNSTON, H.E., CARTWRIGHT, R.A. & WATERHOUSE, J.A.H. (1990). The Inter-Regional Epidemiologic Study of Childhood Cancer (IRESCC): case-control study of children with central nervous system tumors. *Br. J. Neurosurg.*, **4**, 17-26.

BRESLOW, N.E. & DAY, N.E. (1980). *Statistical Methods in Cancer Research*, Vol. I, *The Analysis of Case-Control Studies* (IARC Scientific Publications No. 32). International Agency for Research on Cancer, Lyon.

BROSS, I.D.J. & NATARAJAN, N. (1974). Risk of leukemia in susceptible children exposed to preconception, in utero, and postnatal radiation. *Prev. Med.*, **3**, 361-369.

BUCKLEY, J.D., SATHER, H., RUCCIONE, K., ROGERS, P.C., HAAS, J.E., HENDERSON, B.E. & HAMMOND, G.D. (1989). A case-control study of risk factors for hepatoblastoma: a report from the Children's Cancer Study Group. *Cancer*, **64**, 1169-1176.

BURCH, P.R.J. (1970). Prenatal radiation exposure and childhood cancer. *Lancet*, **64**, 1189.

CARTWRIGHT, R.A., MCKINNEY, P.A., HOPTON, P.A., BIRCH, J.M., HARTLEY, A.L. & MANN, J.R. (1984). Ultrasound examinations in pregnancy and childhood cancer. *Lancet*, **ii**, 999-1000.

CHICCA, M., MUZZOLI, M. & PINAMONTI, S. (1991). In vitro production of free oxygen radicals induced by pulsed ultrasound in whole blood exposed to diagnostic frequencies and intensities. *Boll. Soc. Ital. Biol. Sper.*, **67**, 31-38.

DIAMOND, E.L., SCHMERLER, H. & LILIENFELD, A.M. (1973). The relationship of intra-uterine radiation to subsequent mortality and development of leukemia in children. *Am. J. Epidemiol.*, **97**, 283-313.

DOIDA, Y., MILLER, M.W., COX, C. & CHURCH, C.C. (1990). Confirmation of an ultrasound-induced mutation in two in vitro mammalian cell lines. *Ultasound Med. Biol.*, **16**, 699-705.

GARDNER, M.J., HALL, A.J., DOWNES, S. & TERRELL, J.D. (1987*a*). Follow up study of children born elsewhere but attending schools in Seascale, West Cumbria (school cohort). *B. Med. J.*, **295**, 819-822.

GARDNER, M.J., HALL, A.J., DOWNES, S. & TERRELL, J.D. (1987*b*). Follow up study of children born to mothers resident in Seascale, West Cumbria (birth cohort). *Br. Med. J.*, **295**, 822-827.

GARDNER, M.J., SNEE, M.P., HALL, A.J., POWELL, C.A., DOWNES, S. & TERRELL, J.D. (1990). Results of the case-control study of leukaemia and lymphoma among young people near Sellafield nuclear plant in West Cumbria. *Br. Med. J.*, **300**, 423-429.

GILMAN, E.A., KNEALE, G.W., KNOX, E.G. & STEWART, A.M. (1988). Pregnancy X-rays and childhood cancers: effects of exposure age and radiation dose. *J. Radiol. Prot.*, **8**, 3-8.

GRAHAM, S., LEVIN, M.L., LILIENFELD, A.M., SCHUMAN, L.M., GIBSON, R., DOWD, J.E. & HEMPELMANN, L. (1966). Preconception, intrauterine, and postnatal irradiation as related to leukemia. *NCI Monographs*, **19**, 347-371.

HARTLEY, A.L., BIRCH, J.M., MCKINNEY, P.A., TEARE, M.D., BLAIR, V., CARRETTE, J., MANN, J.R., DRAPER, G.J., STILLER, C.A., JOHNSTON, H.S., CARTWRIGHT, R.A. & WATERHOUSE, J.A.H. (1988). The Inter-Regional Epidemiological Study of Childhood Cancer (IRESCC): case-control study of children with bone and soft tissue sarcoma. *Br. J. Cancer*, **58**, 838-842.

HOWE, G.R., BURCH, J.D., CHIARELLI, A.M., RISCH, H.A. & CHOI, B.C.K. (1989). An exploratory case-control study of brain tumors in children. *Cancer Res.*, **49**, 4349-4352.

JABLON, S. & KATO, H. (1972). Studies of the mortality of A-bomb survivors. 5. Radiation dose and mortality 1950-70. *Radiation Res.*, **50**, 649-698.

KACZMAREK, R.G., MOORE, R.M. Jr, KEPPEL, K.G. & PLACEK, P.J. (1989). X-ray examinations during pregnancy: National Natality Surveys, 1963 and 1980. *Am. J. Public Health*, **79**, 75-77.

KINLEN, L.J. (1993). Can paternal preconceptual radiation account for the increase of leukaemia and non-Hodgkin's lymphoma in Seascale? *Br. Med. J.*, **306**, 1718-1721.

KINNIER WILSON, L.M. & WATERHOUSE, J.A.H. (1984). Obstetric ultrasound and childhood malignancies. *Lancet*, **ii**, 997-999.

MILLER, D.L., REESE, J.A. & FRAZIER, M.E. (1989). Single strand DNA breaks in human leukocytes induced by ultrasound in vitro. *Ultrasound Med. Biol.*, **15**, 765-771.

MOLE, R.H. (1990). Childhood cancer after prenatal exposure to diagnostic x-ray examination in Britain. *Br. J. Cancer*, **62**, 152-160.

MONSON, R.R. & MACMAHON, B. (1984). Prenatal x-ray exposure and cancer in children. In *Radiation Carcinogenesis: Epidemiology and Biological Significance*, Boice, J.D. & Fraumeni, J.F. (eds). pp. 97-105 Raven Press: New York.

NATIONAL CANCER INSTITUTE (1992). *Cancer Statistics Review, 1973-1989*. DHHS Publication No. (NIH) 92-2789. US Department of Health and Human Services, PHS, NIH, NCI: Bethesda, MD.

NISHI, M. & MIYAKE, H. (1989). A case-control study of non-T cell acute lymphoblastic leukemia of children in Hokkaido, Japan. *J. Epidemiol. Comm. Health*, **43**, 352-355.

SHU, X.O., GAO, Y.T., BRINTON, L.A., LINET, M.S., TU, J.T., ZHENG, W. & FRAUMENI, J.F. Jr (1988). A population-based case-control study of childhood leukemia in Shanghai. *Cancer*, **62**, 635-644.

STEWART, A., WEBB, K., GILES, D. (1956). Malignant disease in childhood and diagnostic irradiation in utero. *Lancet*, **ii**, 447.

STEWART, A., WEBB, K. & HEWITT, D. (19580. A survey of childhood malignancies. *Br. Med. J.*, **1**, 1495-1508.

STILLER, C.A. & DRAPER, G.J. (1982). Trends in childhood leukaemia in Britain, 1968-78. *Br. J. Cancer*, **45**, 543-548.

UNITED NATIONS (1986). *Genetic and somatic effects of ionizing radiation. United Nations Scientific Committee on the Effects of Atomic Radiation 1986 Report to the General Assembly, with annexes*. United Nations Sales Publication No. E.86.IX.9. United Nations: New York.

URQUHART, J.D., BLACK, R.J., MUIRHEAD, M.J., SHARP, L., MAXWELL, M., EDEN, O.B. & JONES, D.A. (1991). Case-control study of leukaemia and non-Hodgkin's lymphoma in children in Caithness near the Dounreay nuclear installation. *Br. Med. J.*, **302**, 687-692.

VAN HOFF, J., SCHYMURA, M. & CURNEN, M.G.M. (1988). Trends in the incidence of childhood and adolsecent cancer in Connecticut, 1935-1979. *Med. Pediatr. Oncol.*, **16**, 78-87.

WANG, J.X., INSKIP, P.D., BOICE, J.D. Jr, LI, B.X., ZHANG, J.Y., FRAUMENI, J.R. Jr (1990). Cancer incidence among medical diagnostic X-ray workers in China, 1950 to 1985. *Int. J. Cancer*, **45**, 889-895.

YOSHIMOTO, Y. (1990). Cancer risk among children of atomic bomb survivors: a review of RERF epidemiologic studies. *JAMA*, **264**, 596-600.